# O esporte, a saúde e a vida bancária: a prática do futebol pelos trabalhadores bancários paulistanos, 1929-1932

**DOI:** 10.1590/S0104-59702023000100054

**Published:** 2023-10-23

**Authors:** Gabriela Marta Marques de Oliveira, Edivaldo Góis

**Affiliations:** i Programa de Pós-graduação em História Comparada Universidade Federal do Rio de Janeiro Rio de Janeiro RJ Brasil gbrl.moliveira@gmail.com Doutoranda, Programa de Pós-graduação em História Comparada/Universidade Federal do Rio de Janeiro. Rio de Janeiro – RJ – Brasil gbrl.moliveira@gmail.com; ii Programa de Pós-graduação em Educação Universidade Estadual de Campinas Campinas SP Brasil gois@unicamp.br Professor adjunto, Programa de Pós-graduação em Educação/Universidade Estadual de Campinas.Campinas – SP – Brasil gois@unicamp.br

**Keywords:** História, Esportes e saúde, Saúde dos trabalhadores, History, Sports and health, Worker health

## Abstract

O trabalho analisa a maneira como o futebol se inseriu na vida dos bancários da cidade de São Paulo entre 1929 e 1932. Para isso, trata de quem eram esses trabalhadores e como eles davam sentido às práticas esportivas na Liga Bancária de Esportes Atléticos. Foram utilizadas como fontes relatórios, documentos e publicações da Associação dos Funcionários de Bancos do Estado de São Paulo. As reflexões sobre “classe” e “cultura de classe”, o conceito de “experiência” e a concepção de “classe média” foram utilizados na análise das fontes, na qual se observou variação entre as representações sobre o esporte e as práticas de futebol dos bancários.

## Abstract (en)

This study investigates how soccer was part of the lives of bank workers in the city of São Paulo between 1929 and 1932, looking at who these workers were and the meaning they attributed to sports in the Banking Athletic Sports League (Liga Bancária de Esportes Atléticos). Sources included reports, documents, and publications of the São Paulo State Bank Employees’ Association. Reflections on “class” and “class culture,” the concept of “experience,” and the notion of “middle class” were all utilized in analyzing the sources, and revealed variations in representations of sports and soccer among these employees.

Hoje, já não se pode julgar um povo no seu grau de civilização sem se observar a sua vida esportiva (Cordeiro, out. 1931, p.5).

O objetivo deste estudo foi analisar como o futebol se inseriu na vida de bancários da cidade de São Paulo entre 1929 e 1932, o que exigiu uma compreensão sobre quem eram esses trabalhadores e como eles davam sentido às práticas esportivas na Liga Bancária de Esportes Atléticos (LBEA).

As escolhas para a delimitação deste estudo são respaldadas por uma análise social do Brasil daquele período, na qual é possível perceber como as atividades esportivas eram difundidas em meio à circulação de discursos associados à saúde. As primeiras décadas do século XX apresentam um aumento substancial no número de colunas esportivas nos jornais, que, aos poucos, vão ganhando páginas inteiras dedicadas ao tema. Outrossim, as pautas jornalísticas que abordavam a saúde nas questões da urbanização, da higiene e de práticas consideradas saudáveis ganhavam visibilidade em São Paulo e nos periódicos urbanos.

Certamente essa disseminação dos esportes, em particular do futebol, não era uma característica exclusiva da cidade de São Paulo. Ao contrário, tratava-se de um processo transnacional que, segundo historiadores do esporte, serviu para o estabelecimento não apenas de uma agenda cultural no âmbito mais amplo do imperialismo britânico, mas também como um concurso social que promovia as elites de cidades como Roma, Paris, Nova York e Londres. Nesses contextos citadinos, as classes burguesas disputavam o protagonismo dessas práticas esportivas, que fascinavam os praticantes e os seus públicos. Assim, o império britânico foi um duradouro exportador de futebol, remo, ciclismo e turfe para várias cidades em uma perspectiva global (Mangan, 1986; Holt, 1989), e São Paulo foi um de seus importadores na passagem do século XIX para o XX (Góis Junior, Lódola, Dyreson, 2015).

No Brasil, essas disputas entre as elites pelo controle do esporte também eram patentes, como revelam os estudos de Drumond (2006) que abordaram os conflitos entre grupos distintos da elite carioca em relação ao debate sobre profissionalismo e amadorismo na década de 1930 no Rio Janeiro; de Peters (2014) e Franzini (2003) sobre as disputas entre as elites paulista e carioca em relação à liderança das competições esportivas no país já nas primeiras décadas do século XX; e também de Gambeta (2015) sobre o reconhecimento do futebol como uma prática distintiva no seio das elites paulistanas.

Nos anos 1920 e 1930 no Brasil, essas práticas ganhavam novos sentidos em um contexto cultural, social e político que passava por transformações. Conforme explicam Herschmann e Pereira (1994), há agora uma necessidade premente de se pensar o país em termos nacionais, ou seja, a desejada “modernidade” não poderia ser uma cópia da europeia, pois ela precisava ter mais tons de uma identidade nacional em particular. Falamos de uma identidade nacional que se diferenciava de outras construções simbólicas patentes desde o século XIX no Brasil, porque, como alude Lucia Lippi Oliveira (1997), nos anos 1920, ressaltava-se uma identidade nacional marcada pela crítica aos valores europeus em decadência, depois da derrocada da *Belle Époque* e da Primeira Guerra Mundial.

Diferentemente do período anterior, entre o fim do século XIX e as primeiras décadas do século XX, no qual havia uma persistência pretensamente impositiva de padrões europeus de civilidade, a produção cultural brasileira nos anos 1920 e 1930 buscava uma “modernidade nativa” (Herschmann, Pereira, 1994). Nessa perspectiva, Wood (2019) compreende que, por exemplo, após a Semana de Arte Moderna de 1922, várias das principais figuras literárias da capital paulista recorreram ao futebol em suas criações, buscando desenvolver um novo modelo de brasilidade, mas que no geral não vislumbravam no futebol um exemplo modernista pertinente. Dessa forma, apenas a prática popular do esporte poderia dar vazão a uma reinterpretação antropofágica, pois só se “faz possível à cultura popular brasileira tomar para si a cultura colonizante, reinventando-a sob um viés distinto e imprimindo-lhe uma outra configuração civilizatória – como acontece, justamente, com o destino do futebol inglês no Brasil” (Wisnik, 2008, p.181). Esse papel central dado ao futebol certamente não foi consenso entre cronistas, literatos, médicos e educadores, o que gerou críticos e defensores da prática.

Holanda (2004), por exemplo, ressalta que havia um entendimento de que o futebol, para alguns literatos, era um fator de alienação. Pensavam dessa forma escritores como Oswald de Andrade, nos anos 1930, e, antes dele, Lima Barreto e Graciliano Ramos, na década de 1920, embora outros, como José Lins do Rêgo, tenham contribuído para a construção de uma identidade nacional associada ao futebol.

Por outro lado, como relata Maurício Parada (2003), imperava concomitantemente no período – com a vitória de Getúlio Vargas em 1930 e o Estado Novo a partir de 1937 – uma concepção de educação cívica autoritária que se impunha por meio de datas comemorativas, desfiles, cantos e de uma educação física impregnada pelo disciplinamento da escola. Nesses anos, em São Paulo, observa-se que institucionalmente a “educação física”, com letras maiúsculas, era mais pautada em discursos higienistas e eugênicos de médicos e educadores do que em práticas mais identificadas com o popular ou com os usos do esporte pelas classes populares (Dalben, Góis Junior, 2018).

No momento no qual a educação e a saúde são articuladas como relevantes políticas para o projeto moderno de país (Carvalho, 1998), os esportes não poderiam ser vistos apenas como divertimentos, já que na perspectiva de higienistas a prática esportiva “poderia ser útil para a promoção da saúde, higiene, disciplina, uma ferramenta que poderia contribuir com o progresso do país” (Melo, 2021, p.579).

No que se refere à década de 1930, são muitos os estudos que abordam a necessidade de projetos nacionais de educação e saúde para a conformação da sociedade brasileira, em um tempo no qual médicos higienistas e educadores escolanovistas (Lima, Hochman, 1996; Carvalho, 1998) encontraram um ponto em comum no incremento de uma educação física como disciplina escolar com objetivos de formação de um homem nacional (Parada, 2003). Naquele momento, tratava-se da defesa de uma “eugenia positiva”, baseada principalmente na intervenção do Estado no sentido de uma promoção higienista da educação e da saúde, pois a “melhoria da raça” tornava-se sinônimo de oferecer condições apropriadas de saneamento para a população (Lima, Hochman, 1996; Stepan, 2004).

Nesse âmbito, as relações entre esportes e políticas governamentais de saúde e educação são abordadas pela literatura especializada no sentido de observar os objetivos estratégicos do Estado em relação ao controle dessas práticas. Por exemplo, Gomes e Dalben (2011), ao estudarem o Departamento de Educação Física do estado de São Paulo, enfatizaram que na década de 1930 havia a organização de um aparato científico promovido pelo governo paulista que desejava balizar uma educação física orientada segundo os conhecimentos da medicina. Da mesma forma que se reivindicavam um sistema público de educação e uma intervenção estatal no campo da saúde. O médico Arthur Neiva, em São Paulo, pressionava por uma maior participação do Estado na organização dos esportes (Dalben, Góis Junior, 2018).

O futebol, entretanto, era mencionado nos jornais mais como um divertimento do que uma prática saudável, pois talvez não estivesse suficientemente ligado, a despeito dos desejos e discursos de médicos e educadores, aos projetos nacionais. Houve, inclusive, produções de médicos que eram céticas em relação aos benefícios do esporte na educação e saúde, como a de Carlos Sussekind de Mendonça, em 1921 (Dalben, Góis Junior, 2018). O futebol, entretanto, era o esporte mais difundido nos jornais e, por isso, por meio de sua visibilidade, era relevante para os higienistas colocarem em curso a sua conformação e fiscalização.

Nesse sentido, a década de 1930, para Drumond (2006), representou um maior avanço do Estado, em particular na política varguista de “oficialização” dos esportes e na organização de instituições de controle, como a Comissão Nacional de Desportos e o Conselho Nacional de Desportos. Em São Paulo, esses objetivos foram primeiramente colocados pela criação do Departamento de Educação Física, em 1931, como resultado de um debate sobre a necessidade de se organizar e controlar a prática esportiva (Dalben et al., 2019). Para além dos divertimentos, o futebol e os esportes, na perspectiva higienista, teriam que ter relevância por sua possibilidade de incremento do vigor físico da nação (Pereira, 2000; Souza, 2008).

Essa educação física marcada por uma moral cívica pautada por comportamentos aceitos e recomendados por médicos e educadores em uma perspectiva de saúde não era exclusiva de São Paulo ou do Rio de Janeiro. A literatura especializada internacional também reforça essas expectativas sobre os esportes na educação de jovens e trabalhadores (Holt, 1989; Mangan, 1986; Rabinbach, 1992). Como se dava, contudo, a experiência dos trabalhadores em meio a essas práticas, mesmo diante dos interesses de intelectuais, médicos e educadores favoráveis e contrários aos esportes?

Na impossibilidade de construção de uma interpretação genérica sobre a perspectiva dos trabalhadores na prática esportiva, optamos pelo estudo de uma categoria profissional em particular: os bancários. Desse modo, pela originalidade das fontes sobre uma perspectiva sindical em relação aos esportes no período, este estudo pretende contribuir sobre outras formas de apreensão da prática esportiva e, em especial, do futebol.

## Os caminhos da pesquisa entre esporte, política e associativismo

Nos jornais, sobretudo na cidade de São Paulo, os cronistas esportivos noticiavam os acontecimentos e resultados dos jogos, principalmente daqueles realizados pela Liga Amadora de Futebol (LAF), Liga Paulista de Futebol (LPF) e Associação Paulista de Esportes Atléticos (Apea). Os jogos não vinculados a associações e ligas esportivas tinham maior ou menor destaque na mídia, a depender do jornal e de quem eram os sujeitos que os praticavam. Analisando esses periódicos, nos deparamos com a LBEA, vinculada à Associação dos Funcionários de Bancos do Estado de São Paulo.

Ela foi fundada em 16 de abril de 1923, por Francisco Silva Pinto, funcionário do City Bank. O intuito da liga era o de ser uma associação assistencialista. Oferecia médico, enfermaria, dentista, serviço de farmácia, curso de contabilidade, departamento de colocações e serviços jurídicos. Tentava-se promover a identificação entre os bancários (Canedo, 1977). Vejamos como os esportes contribuíram para essa identificação de classe, e como eram encarados pela associação e pelos funcionários bancários.

Nesta pesquisa, analisamos o momento inicial de inserção do esporte, em particular, do futebol, nas páginas do jornal oficial da Associação dos Funcionários de Bancos do Estado de São Paulo, entre 1929 e 1932, mediante as diversas apropriações daquela prática por parte desses trabalhadores, que, por vezes, oscilavam entre o divertimento e os seus usos utilitários voltados para a saúde. Esse recorte temporal justifica-se, porém, não somente pelas primeiras aproximações daquela associação de trabalhadores bancários com as práticas esportivas, mas por ser um momento no qual a organização daquele sindicato parecia reproduzir discursos higienistas que circulavam entre as classes médias e propalavam os benefícios saudáveis da prática esportiva, a partir de um sistema de valores que reivindicava o lugar de enunciador das boas condutas. Além disso, é importante salientar que o recorte se insere na primeira fase de vida da associação *–* que vai de 1923 a 1932 – baseando sua organização na assistência prestada aos associados (Canedo, 1977). Assim, a visão de esporte adotada e transmitida por ela pode ser compreendida como mais uma forma de assistência aos seus membros não diretamente relacionada com o mundo do trabalho, mas com a saúde e o “bem-estar”.

Nessa interrogação às fontes, formaram o corpo documental, principalmente, os relatórios da associação e o jornal mensal *Vida Bancária*, pois ele era o órgão oficial da associação entre sua fundação (1923) e setembro de 1939. Em 1931, a associação muda seu nome para Associação dos Bancários de São Paulo – Órgão Sindical.

Entre 1929 e 1932, o jornal manteve certa regularidade com edições mensais, totalizando 43 números publicados nesse período. Cada fascículo tinha de quatro a oito páginas, e o jornal alcançou a tiragem de cinco mil exemplares em 1932. Era um veículo privilegiado de comunicação entre os dirigentes da associação e os trabalhadores bancários, refletindo um esforço de arregimentação e uma preocupação de doutrinar no “ideal sindicalista” (Canedo, 1977).

A análise documental se deu a partir da leitura integral dos números do *Vida Bancária* publicados no período pesquisado. A partir dela, dois movimentos foram possíveis: (1) ter uma visão geral de quais eram as discussões existentes dentro da associação, quais as reivindicações desses trabalhadores, e qual a visão de sua situação de classe; e (2) selecionar os artigos que discutiam, especificamente, a prática esportiva e as questões de saúde entre os bancários. Uma tabela com ano, número, página, título, autor, grande tema e resumo foi produzida, contendo cada um dos artigos do jornal. Após a primeira leitura, de 451 artigos, foram encontrados 116 que tratavam do tema. Eles foram analisados a partir da perspectiva da história social inglesa, notadamente das reflexões de E.P. Thompson (1981, 1998, 2012) sobre “classe” e “cultura de classe”, e do conceito de “experiência”.

Como órgão oficial, o *Vida Bancária* era o responsável por promover a liderança dos dirigentes da associação, divulgar suas ações, projetos e, sobretudo, as concepções sobre o trabalho dos bancários. Em suas páginas, é possível observar a organização de uma associação que se destaca pelo assistencialismo. Em particular, no período estudado, tanto a associação quanto a linha editorial do *Vida Bancária* esmeravam-se nas tentativas de sistematização de ações assistencialistas, com o provimento dos serviços já citados. Houve, em 1929, uma proposta de entregar o jornal a pessoas estranhas à diretoria da associação, mas o projeto não foi adiante. Em 1932, *Vida Bancária* passa a circular com características de revista e circulação restrita (Canedo, 1977). Não é possível definir quem eram os autores dos textos publicados no jornal, uma vez que, em sua maioria, eles eram publicados sob pseudônimos. No entanto, a seção esportiva tinha como redator sempre um membro da diretoria da liga.

Também na questão esportiva, havia a intenção de promover e disponibilizar um serviço aos bancários. Segundo as linhas do *Vida Bancária*, entretanto, nos termos de L.C. (fev. 1929, p.4), o esporte não era uma das práticas mais concorridas entre os bancários, mas aquele quadro deveria mudar, pois a educação física seria uma questão de importância central, uma vez que contribuiria para a manutenção da saúde. Ele ressalta o utilitarismo do esporte como ferramenta compensatória para o desgaste propiciado pelo trabalho. Seria, então, preciso promovê-lo como iniciativa de higiene e saúde, preparando o trabalhador para uma árdua jornada. Sem dúvida, essa poderia ser uma apropriação do esporte por parte da parcela dos bancários que dirigia a associação, que atuava mais como uma entidade de assistência do que uma organização sindical. Havia, no entanto, outras apropriações do esporte no âmbito da estruturação dessa categoria profissional. Enfim, quem seriam esses bancários? Seria possível uma definição mais ampla desses trabalhadores? E como eles se inseriam no universo político-social e esportivo da cidade?

Em 1929, após a posse da nova diretoria, o presidente da associação foi procurado por representantes do London Bank Club com a proposta de criação de uma liga esportiva, que se dedicaria apenas ao futebol, “o único esporte que tem tido um certo incremento entre os bancários” (L.C., fev. 1929, p.4). Uma reunião com representantes dos clubes bancários foi marcada para se discutir o tema na associação. No dia marcado, representantes dos clubes dos bancos Francês e Italiano, Banco de Minas, British Bank, London Bank, Banco Comercial, Casa Bancária Conde e Almeida, Royal Bank e Banco Comércio e Indústria compareceram à sede da associação. A criação da LBEA foi aprovada por sete desses clubes, sendo contrário apenas o representante do Banco Comércio e Indústria. O princípio dos estatutos seria o seguinte: todo jogador inscrito na liga deveria ser sócio da associação.

Nova reunião ficou marcada para se decidir sobre a organização definitiva da LBEA, de seus estatutos e da eleição da diretoria. A notícia da necessidade de filiação à associação para tomar parte na liga, no entanto, repercutiu negativamente nos bancos. A associação, por sua vez, argumentava que essa cláusula havia sido aprovada pelos presentes, não se podendo atribuir a imposição de tal regra a ela. Decide-se, então, por patrocinar a LBEA sem a necessidade de filiação dos jogadores à associação.

A fundação de uma liga de futebol sob o patrocínio da associação de classe dos bancários estava em consonância com o pensamento de lideranças comunistas do período, que apoiavam a chamada “proletarização dos esportes”. O que se pretendia com isso era a tomada para si, por parte dos sindicatos e das associações, da organização do esporte dos trabalhadores. Isso porque essas lideranças acreditavam que o futebol, “esporte burguês”, estava desviando as atenções dos trabalhadores, tirando deles o foco na luta por melhores condições de trabalho e de vida. Após anos de negação do futebol como parte da cultura dos trabalhadores, passou-se a defender a prática desse esporte nos sindicatos. Entendia-se que, caso eles não tivessem contato com o futebol dentro dos sindicatos, o teriam fora deles, então seria melhor para a luta operária que esses trabalhadores pudessem praticar os seus esportes sob o patrocínio de seus sindicatos. Fato é que, nesse momento em que lideranças comunistas começaram a olhar para o futebol como um “mal necessário” a ser cultivado dentro dos sindicatos, a associação criou sua própria liga, que congregava clubes de diversos estabelecimentos bancários. Porém, o fato de que os jogadores não precisavam ser filiados à associação para tomar parte nos campeonatos pode indicar que, ao contrário do que acontecia em outros contextos – como o da juventude comunista do Rio de Janeiro dos anos 1930 (Góis Junior, Soares, 2018), da Alemanha com a Socialist Workers’ Gymnastics Federation (Krüger, 2014) ou da Inglaterra com a British Workers’ Sports Federation (Jones, 1985) –, não havia ali uma tentativa explícita de arregimentar sócios para a associação por meio da prática esportiva ou de promover a causa operária por meio da educação física.

Ainda no período analisado houve a filiação da LBEA à Apea. Ela era uma das duas associações responsáveis pelo esporte na cidade de São Paulo naquele momento. Esse acontecimento pode nos dar indícios de pelo menos três coisas: (1) a LBEA tinha alguma relevância nos meios esportivos paulistanos, uma vez que fora reconhecida pela máxima instituição esportiva do estado; (2) havia, por parte dos esportistas bancários, uma busca pela institucionalização de sua prática esportiva, e o respaldo da Apea era o primeiro passo a ser dado nesse sentido; e (3) a Apea tinha a intenção de controlar o máximo possível o esporte paulistano, já que congregava em torno de si outras associações esportivas menores. Era interessante para essas associações, uma vez que conseguiam o respaldo da principal organizadora do esporte paulistano, e era interessante para a Apea, que adquiria o monopólio da organização esportiva, tornando-se grande o suficiente para superar sua rival, a LPF.

No que se refere ao mundo futebolístico paulistano, 1929 ficou marcado pela unificação das duas ligas que comandavam o futebol na cidade – a LAF e a Apea. O Clube Atlético Paulistano – fundador de ambas as ligas – decide abandonar a LAF e fechar o seu departamento de futebol quando a discussão sobre a profissionalização desse esporte começa a ficar mais séria. A partir daí, até meados da década seguinte, a discussão sobre o profissionalismo seria ponto de destaque entre cronistas esportivos.

Havia grande resistência dos clubes em regulamentar a profissionalização do jogo, que, no entanto, passa a ser apoiada por boa parte dos cronistas esportivos da época, por diversos motivos. Um deles seria a necessidade de se impedir a evasão dos bons jogadores paulistas, que estavam sendo levados por times europeus que já adotavam o futebol profissional (Lopes, 2004). Outro motivo foi a prática do falso amadorismo, que consistia no pagamento de recompensas – conhecido como “bicho” – aos jogadores pelo seu desempenho e pelo resultado do time no jogo. A imprensa defendia que, com o profissionalismo institucionalizado, os clubes teriam que pagar aos jogadores um preço mais justo (Caldas, 1988). Apesar disso, muitos dos clubes e cronistas que se colocavam contrários ao profissionalismo no futebol paulista o faziam reivindicando questões relacionadas ao espírito esportivo e ao amadorismo puro, defendendo o esporte apenas como forma de deleite e de treinamento do corpo, seguindo as visões higienistas que eram fortes no período. Esse pensamento tinha eco, também, dentro do esporte bancário, como demonstra o artigo de Cordeiro (nov. 1931, p.3-4) sobre as “finalidades do esporte”:

O esporte deve ser cultivado para a saúde do corpo e do espírito; aplicar na vida prática os benefícios que ele proporciona.

Não é só cultivá-lo fanaticamente, vivendo quase que exclusivamente para ele. Que vivam para ele, e dele, os profissionais, está muito certo. Mas um amador?, não.

O esporte, cultivado assim cegamente, traz como consequência o empedernecimento do cérebro. Conseguem enrijecer os músculos, mas tornam o cérebro embrutecido ou rústico; conseguem eficiência e admiração, mas, às vezes, consequências desagradáveis.

Um indivíduo que só cuida de esporte é um indivíduo nulo para a coletividade: já pelo espírito quase obtuso, já pela indiferença com que encaram os outros magnos problemas da coletividade, muitas vezes com desconhecimento completo.

Pratique-se o esporte na sua verdadeira finalidade, traduzindo na moral, no caráter, no trabalho, na vida coletiva o revigoramento que ele dá ao corpo. O esporte é indispensável, mas, moderado, bem compreendido e melhor aplicado.

No plano nacional, as transformações políticas ocorridas durante as décadas de 1920 e 1930 ajudam a compreender o momento pelo qual o país passava. Era uma fase de transição política em que se concretizariam, a partir do movimento de 1930, a centralização da política e a intervenção do Estado nas questões sociais e trabalhistas. Aconteceram também ampliações dos setores urbanos e das camadas médias que impulsionariam alterações no campo político, como “o questionamento das bases do sistema oligárquico da Primeira República” (Ferreira, Pinto, 2017, p.390), fomentando disputas regionais e afetando as eleições presidenciais. Um cenário político que teve como desfecho a deposição de Washington Luís e a vitória de Getúlio, dando início à Era Vargas.

Esse é um período repleto de acontecimentos políticos e sociais importantes. Já em 1931 tem-se a Lei de Sindicalização, com o objetivo de combater organizações que permanecessem independentes (Gomes, 2005). O decreto-lei foi muito bem recebido pelos bancários, que entendiam o controle do Estado como benéfico naquele momento (Canedo, 1977). A partir de 1933-1934, o Estado vinculou o gozo dos benefícios sociais à condição de trabalhador sindicalizado, o que pode ajudar a explicar o aumento no número de sindicalizados. Como forma de resistência, as lideranças de esquerda decidem tomar parte dos sindicatos oficiais. Apesar de reconhecerem a impossibilidade de desprezar os benefícios sociais, entendiam ser fundamental resistir à proposta corporativista tanto na Constituinte quanto nos sindicatos e nas ruas (Gomes, 2005). Dessa forma, a experiência dos membros da associação está ligada às diversas disputas políticas inerentes à sua existência.

Fica claro, a partir do exposto, que 1929 foi marcante para a associação, com a fundação da LBEA, com o início das movimentações que levariam ao golpe de 1930 e com os conflitos no cenário futebolístico da cidade de São Paulo. Foi o momento em que houve o fechamento do time de futebol do C.A. Paulistano, quando as discussões sobre o profissionalismo no futebol se avolumaram. A partir de 1933, no entanto, tem-se uma mudança na autocompreensão dos bancários, que tornaram o sindicato mais combativo politicamente, o que vai impactar a visibilidade das práticas esportivas em suas publicações periódicas (Canedo, 1977; Oliveira, 2020). Por isso, é relevante discorrer sobre a “identidade profissional” (Dubar, 2005) dos bancários para compreender suas práticas.

## Os “operários da casaca”

Os bancários formavam uma categoria profissional bastante numerosa nos anos 1930. Em 1931, um levantamento realizado pela associação mostrou que havia na cidade 2.629 bancários (Estatística..., jul. 1931, p.2), sendo que 1.459 dela faziam parte (R.R., set. 1931, p.1). Eles eram divididos entre escriturários e contínuos. Os primeiros trabalhavam na administração do banco. Eram gerentes, subgerentes, contadores, procuradores, escriturários, caixas, correntistas etc. Suas funções estavam ligadas às atividades-fim dos bancos. Já os contínuos eram funcionários do quadro de portaria. Eram porteiros, vigias, ascensoristas, telefonistas etc. e tinham funções ligadas às atividades-meio das agências.

O modo como se viam, como mais ou menos próximos de outras categorias de trabalhadores variava, mas, durante todo o período analisado, eles se enxergavam como trabalhadores. Eram, em suas palavras, “operários da casaca” (O presidente..., mar. 1931, p.1), que tinham condições de trabalho ruins e recebiam pouco pelo trabalho realizado e para a manutenção do padrão de vida que deveriam aparentar ter. Pela sociedade, porém, eram vistos como uma classe privilegiada, já que recebiam mais do que a média salarial da cidade e pareciam sustentar um bom padrão de vida.

Segundo Williams (2007), com o desenvolvimento das funções de escritório houve uma ambiguidade no que se refere à posição dos que trabalhavam por salário, mas não realizavam trabalho manual. Eles, muitas vezes, foram caracterizados como “classe média”. A locução, referindo-se àqueles que ganham salários, é “uma expressão de posição social relativa e, portanto, de distinção social” (p.92). Já “classe trabalhadora”, a partir de uma noção de classe produtiva, é uma expressão de relações econômicas. As duas expressões, desse modo, apoiam-se em modelos diferentes. Um grupo, ao se definir como classe média, mostra ter consciência de sua posição social relativa, de certa distinção social; porém, quando esse mesmo grupo se encontra no interior de uma relação econômica em que depende de seu trabalho, há um ponto crítico de sobreposição dos termos, podendo haver confusão entre eles.

Mills (1979), ao analisar a formação da nova classe média nos Estados Unidos, no início do século XX, olha para questões não apenas econômicas, mas também psicológicas e sociais. Segundo ele, constituem a chamada “nova classe média” americana funcionários que recebem seu salário por mês; que conseguem algum *status* social, a partir de seu trabalho; e que utilizam roupas de passeio no local de trabalho. As três características apresentadas se complementam para a formação do que ele entende por um funcionário de “colarinho branco”. O *status* social que consegue por meio do trabalho estaria ligado ao fato de receber por mês e, portanto, de conseguir se diferenciar de outro tipo de trabalhador, o que recebe por dia ou hora. Ainda o fato de utilizar roupas de passeio traz a esse trabalhador mais *status*, já que, novamente, não será confundido com o operário. No entanto, a possibilidade de utilização de vestimenta de passeio só é possível pelo exercício de uma profissão que garante *status* social. É uma via de mão dupla. Esses trabalhadores de “colarinho branco” passaram a ser vistos pela sociedade de maneira diversa de outros tipos de trabalhadores, mas também se enxergavam como diferentes deles, com uma identidade profissional própria. Eles tinham outros anseios, outras perspectivas e outro *status*.

Owensby (1999), ao analisar a formação da classe média no Brasil entre os anos 1920 e 1950, afirma ter havido um esforço das associações de classe dos “colarinhos brancos” em frisar uma fronteira entre eles e os trabalhadores manuais na construção de sua identidade profissional. Teria sido nesse momento, portanto, que essas associações localizaram esses funcionários em uma nova taxonomia de classe, a classe média (Owensby, 1999). No que se refere à associação dos bancários, no entanto, é difícil fixar o momento em que essa fronteira começa a ser delineada. Ao contrário, o que verificamos é a existência de uma linha tênue entre a vinculação com trabalhadores manuais e com trabalhadores não manuais. Ela dependia, em muitos casos, de qual era a reivindicação da categoria e de que maneira essa reivindicação era feita. Um exemplo disso pode ser a negociação diretamente com os bancos pelas duas horas de almoço em 1932, e a realização da greve de 1934, em defesa da criação do Instituto de Aposentadoria e Pensões dos Bancários.

Os salários variavam muito de banco para banco e dentro do próprio banco, como é possível constatar nas tabelas feitas pela associação, em meados dos anos 1930^1^ e nos diversos artigos que tratam das condições de vida dos bancários. Em um número do *Vida Bancária* de 1942, tratando da composição salarial média dos bancários em 1935 e 1941, temos as seguintes cifras:^2^

**Quadro 1:** : Média salarial nos bancos

Bancos	1935	1941	Acréscimo
Um grande banco nacional	612$000	934$000	52%
Um pequeno banco nacional	532$000	631$000	16%
Um grande banco estrangeiro	950$000	1:398$000	47%
Um pequeno banco estrangeiro	415$000	864$000	108%

Fonte: Memolo Netto (jun. 1942, p.12).

Nesse mesmo artigo é apresentada uma estatística feita pelo Ministério da Fazenda sobre o custo de vida no Rio de Janeiro entre 1912 e 1941. Toma-se por média uma família de quatro pessoas, e entende-se que uma família do Rio e uma de São Paulo precisariam do mesmo valor de 1:040$000 para sua subsistência em 1935. Em 1943 o salário-mínimo era de 360 cruzeiros (Operários..., fev. 1945, p.3), e em 1944 o ordenado médio dos bancários era de 700 cruzeiros (Ao lado..., jul. 1944, p.6). Esses números demonstram a dificuldade em definir a média salarial dos bancários e qual o seu padrão de vida; porém, é possível perceber, a partir dos dados apresentados, que se tratava de uma categoria de trabalhadores que ganhava mais do que o salário-mínimo do estado de São Paulo, que, aprovado em junho de 1939, era de 200$000 para a zona da capital (Fixado..., 17 jun. 1939, p.7).

Levando-se em consideração os dados apresentados, podemos concluir que os salários recebidos pelos bancários não eram suficientes para sustentar o padrão de vida médio de uma família de quatro pessoas. E eles diziam isso correntemente. Viam-se como trabalhadores mal remunerados, como é possível perceber na passagem a seguir, escrita por Americano Meridional em junho de 1934 (p.2):

Daí a vida necessitada que passa e que é obrigado a passar, por várias circunstâncias, vejamos: – custo de vida exorbitante, alta dos gêneros, artigos de uso, casa, locomoção, diversões etc. em face dos salários baixíssimos, humilhante[s] e microscópicos que nos são pagos em doses homeopáticas. ... que obrigam os bancários a se tornarem escravos das casas de penhores, dos agiotas, dos títulos, dos protestos, das dívidas insolúveis, das prestações, dos judeus.

Havia uma exigência constante sobre os funcionários de bancos a respeito de sua forma de vestir, sua aparência e seu comportamento.

Obrigados, pela natureza do trabalho e pela sua situação social, a aparentar, apresentando-se decentemente, esses novos mártires da sociedade moderna vivem num círculo cruel de misérias, de necessidades e de humilhações.

Barba feita, roupa limpa e sem remendos, sorrisos para os clientes, boas e custosas amizades, tudo isso tem os companheiros bancários que apresentar numa hipocrisia obrigatória, quando, por trás da máscara dessa felicidade mentirosa, vivem numa luta torturante, atroz, acabrunhadora, com os agiotas, com o desconforto, com o sacrifício inevitável da família, com credores impacientes e mesmo com a fome. Daí, certas doenças, e a desmoralização pelas dívidas, culminando no desemprego, porque aos bancos não fica bem ter funcionários doentes ou sem moral (Miséria..., 14 maio 1935, p.1).

Essas exigências contribuíam para que fossem vistos como parte dos setores privilegiados da sociedade. Tendo sempre que estar muito bem vestidos, gastavam parte considerável de seus salários com roupas. Esta era a maior das queixas: a necessidade de uma aparência sempre impecável, que não condizia com a realidade econômica, mas que ajudava na manutenção de um *status* diferenciado.

Chalhoub (2012) demonstra de que maneira se deu a tentativa de aburguesamento da sociedade carioca no início do século XX. O autor, ao tratar do trabalho, da vida familiar, e dos divertimentos dos trabalhadores, aponta para um projeto político de reforma social que, veiculado pela classe dominante, tinha como objetivo impor mudanças materiais e um novo modo de vida aos trabalhadores da capital federal, inserindo o país na “civilização”. Chalhoub (2012) apresenta diversos exemplos de como esses trabalhadores, apesar da forte investida das classes dominantes, resistiram às suas imposições, fazendo com que a burguesia tivesse que se contentar com um mundo criado à sua imagem imperfeita. Nesse sentido, vai se forjando, a partir do início da República, uma nova ética de trabalho e um modelo de família para os membros da nação.

Popinigis (2007), ao se debruçar sobre a vida e a organização dos trabalhadores do comércio carioca no início da República, argumenta que eles formavam uma categoria de empregados cujas funções eram intelectuais e que eles tinham uma identidade, ainda que dentro dos empregados no comércio houvesse desde os caixeiros de botequins até os empregados de lojas de roupas importadas. A identidade, neste estudo, consiste na compreensão de Claude Dubar (2005) de que ela se constrói nas relações entre o individual e o coletivo, ou seja, emerge de uma socialização que identifica um indivíduo em múltiplas dimensões, inclusive em uma central: a “identidade profissional”. Os bancários, nesse âmbito, construíram sua luta política por meio de uma identidade profissional, apesar de estarem subdivididos em categorias específicas, que tinham particularidades de hierarquia, função, sociabilidade e práticas de divertimento.

As análises de Chalhoub (2012) e Popinigis (2007) são complementares e jogam luz sobre pontos importantes para a nossa pesquisa, uma vez que a tentativa de imposição de um determinado modo de vida, e a forma de organização de trabalhadores – bem como a atuação do Estado nessa organização –, no início do século XX, são parte importante deste trabalho. Havia uma tentativa das classes dominantes – aqui encarnadas nos donos e gerentes de bancos – em enquadrar os trabalhadores bancários em uma nova ética de trabalho, modelo de família e ocupação do tempo de não trabalho. No entanto, essa tentativa não teve êxito completo, na medida em que esses trabalhadores resistiam e ressignificavam essas imposições, constituindo sua “identidade profissional”.

Assim, como os funcionários do comércio analisados por Popinigis (2007), os bancários também formavam uma categoria com cultura e estilo de vida próprios. Eles tinham gostos específicos pelo vestir, pela mobília e decoração, uma forma de organização política específica, preferências por maneiras de se sociabilizar e, é claro, preferências por determinados divertimentos, como veremos no caso do futebol.

## Entre a vida esportiva e a vida bancária

No início de 1929 a LBEA era apenas uma ideia que consistia na defesa da importância de uma organização esportiva para os bancários. L.C. (fev. 1929, p.4) escreve:

A mais simples análise ressalta que, entre os bancários, a propensão para os esportes não é das mais acentuadas. Como que a querer destruir esta asserção, poderiam nos apontar as muitas esquadras de futebolistas que existem pelos bancos, algumas já de antiga fundação. ...

Para os bancários, a educação física é uma questão de capital importância e, como tal, deve ser encarada. Passando longas horas arcados sobre uma escrivaninha, na árdua tarefa de enfileirar algarismos, dispendendo com isso grandes reservas de energia, todo o organismo reclama, depois, a prática salutar dos esportes, para refazer-se dessas perdas de vitalidade. ...

Urge, em nosso meio, mover-se uma intensa propaganda esportiva, concitando os nossos colegas a aderirem aos clubes internos que, com esse fim, já existem em muitos de nossos bancos para que possamos, dentro em pouco, contar com um grande contingente de esportistas. ...

Julgamos imprescindível a criação de uma liga, essencialmente bancaria, à qual os clubes internos ficassem subordinados.

Para ele, os bancários deveriam se juntar aos clubes já existentes para praticar algum exercício físico, que provavelmente seria o futebol – o esporte que fazia sucesso entre eles no período. Eles deveriam fazê-lo para recuperação das energias dispendidas no trabalho. Foi esse o impulsionador da criação da LBEA, em consonância com o ideal higienista que tinha por finalidade tomar ações normativas no campo do trabalho (Rabinbach, 1992) e, particularmente, no campo esportivo brasileiro, propagandear sua prática controlada para “melhorar a raça” (Dalben, Góis Junior, 2018; Silva, 2014).

No caso dos bancários, apesar da pouca competitividade entre os concorrentes do campeonato, as discussões e os incidentes eram recorrentes e não ficavam atrás dos torneios oficiais. Os “casos”, como se referiam os cronistas aos desentendimentos no interior dos campos, avolumavam-se rodada após rodada. Sobre o assunto, Peregrino Memolo Netto (jun. 1929, p.4) escreve: “A educação esportiva em nossa terra ainda está longe de atingir o grau em que ela merecia estar, atendendo-se ao nosso progresso técnico”. Para ele – que era funcionário do London Bank e foi presidente em 1929, suplente da diretoria em 1933, secretário-geral da LBEA em 1935 e membro da diretoria da associação em 1937 e 1938 – os desentendimentos que ocorriam entre os futebolistas bancários tinham ainda menos razão de ser do que os que se davam nos campos do futebol oficial. Isso porque:

Em primeiro lugar, os jogos da liga não têm renda de bilheteria e, por conseguinte, não possuem uma assistência numerosa e partidária, capaz de influir no ânimo do jogador. Em segundo lugar, os elementos que atuam nos campos da liga são elementos de uma mesma classe, absolutamente homogêneos, e que, nós o supomos, não são desconhecedores da educação esportiva (Memolo Netto, jun. 1929, p.4).

Por “educação esportiva” o autor provavelmente entendia os preceitos defendidos pelo amadorismo, que consistiam no cavalheirismo, no respeito ao adversário, no deleite da prática do jogo e no respeito às regras previamente acordadas. Se era possível supor que essa “educação esportiva” era dominada pelos bancários, não se pode dizer o mesmo da “educação física”. Nesse momento havia a necessidade de se ensinar aos trabalhadores bancários sobre os benefícios que a prática de exercícios físicos poderia lhes trazer. Assim, diversas advertências sobre o assunto foram feitas por meio das páginas do *Vida Bancária*. Yapo (jan. 1930, p.11) escrevia que “deitar cedo, levantar cedo o mais possível e não usar nunca o fumo e o mínimo possível as bebidas alcoólicas são as sólidas bases de quem deseja ser um bom atleta”; ou ainda que “todo esportista, à proporção que melhora o seu físico, deve envidar todos os esforços para que as suas qualidades morais também se elevem e se nobilitem”. Yapo, aliás, era um dos maiores propagadores das práticas que se deveria adotar para tornar-se um bom atleta. Ele, em outra edição do jornal, afirmava ser necessário que “abandonemos e combatamos o álcool, o fumo, o jogo e os excessos viciosos que são o cancro horrendo que suga as energias morais e físicas dos nossos jovens patrícios e, portanto, consomem as forças da Nação. Cultivemos o esporte sem excessos e pelo amor ao esporte!” (Yapo, fev. 1930, p.5).

Ao passo que as representações sobre as práticas esportivas eram construídas pelos redatores do *Vida Bancária* em consonância com os dirigentes da associação, e caminhavam na direção de um alinhamento com os códigos morais propalados por médicos e educadores em uma perspectiva de “educação esportiva”, concomitantemente, na direção contrária, as práticas esportivas daqueles trabalhadores bancários ganhavam outros sentidos na busca de uma diversão desinteressada, do júbilo, do prazer, a despeito da moralidade expressa naqueles impressos. Do mesmo modo, Melo e Peres (2016, p.1148) também interpretaram que, por exemplo, os discursos sobre a ginástica nas escolas oitocentistas nem sempre se materializavam em um sentido estrito nas práticas, pois para eles “não devemos negligenciar o fato de que frente a uma proposta de rigidez corporal também se manifestasse um corpo transgressor, que dialogava com outras experiências sociais”.

Nesse sentido, esse discurso adotado pelo órgão oficial da associação não parece ter muita conexão com a realidade das disputas futebolísticas. Um exemplo é o encontro amistoso entre C.E. Induscomio e o E.C. Sudameris, em outubro de 1929, como parte de um convescote bancário. O evento reuniu quase setecentas pessoas, contando bancários e seus familiares, no parque da Vila Galvão, e teve como ponto alto o encontro entre os dois times. Antes do início de um jogo bastante movimentado os quadros se alinharam no gramado para uma troca de gentilezas. O Sudameris ofereceu uma corbélia ao Induscomio, que retribuiu o gesto oferecendo “aos capitães, juízes, representante da liga bancária e diretores de ambos os clubes um delicioso copo de vinho espumante” (Match..., nov. 1929, p.4). O consumo de álcool, demonizado por Yapo, esteve presente antes do início do jogo sem nenhuma cerimônia. Outro caso emblemático para demonstrar o descolamento entre o discurso de ideal esportivo e a prática da LBEA é a cerimônia de premiação do campeonato de 1931.

Realizada em 21 de janeiro de 1932, essa cerimônia contou com a presença de muitos bancários no salão nobre da associação. Na ocasião, o doutor Francisco de Paula Reimão Hellmeister, presidente da liga (e que faria parte da diretoria da associação em 1936), fez um discurso de saudação às equipes vencedoras do campeonato, Induscomio e British Bank. Após os capitães das equipes terem recebido os respectivos troféus, era hora de começar a festa. “Aos presentes foi oferecido grande quantidade de ‘cristalino’ *chopp* Antarctica o que, naturalmente, veio aumentar o seu entusiasmo” (A entrega..., jan. 1932, p.3).

Novamente, contrariando a representação de bons atletas veiculada pelo *Vida Bancária*, o que a liga faz é oferecer bebida alcoólica aos presentes, dessa vez em grande quantidade. Essa prática nos dá indícios de que o ideal higienista de formação de atletas, em que seria preciso cuidar do corpo e da alimentação para se tornar um ser humano moral e fisicamente mais habilitado, não encontrava eco nas práticas dos esportistas bancários do período. Ou seja, a experiência desses sujeitos parecia distante das representações sobre a prática esportiva vigentes naquele momento. Acreditamos que, para eles, mais do que se tornar atletas com bons rendimentos, de corpos sadios e moral elevada, era a utilização do tempo livre na participação em um ambiente de descontração e de congraçamento entre os pares que fazia com que se vinculassem aos clubes e disputassem campeonatos. Eles buscavam a vitória, mas ela não estava necessariamente atrelada a possuir valores atléticos refinados, como se pregava nas páginas do órgão da associação.

A prática futebolística fazia parte da experiência e da cultura dos bancários. É possível, no entanto, perceber, a partir do exposto, que eles resistiam e ressignificavam as ideias propostas pelos seus representantes no órgão oficial de sua associação de classe, de maneira semelhante ao que demostrou Chalhoub (2012), em relação ao projeto de modernidade brasileira. Isso porque as pessoas experimentam a própria experiência não apenas no âmbito do pensamento, mas também como sentimento, como normas e como valores (Thompson, 1981).

Assim, os bancários paulistanos aproveitavam a existência de uma liga de futebol organizada por sua associação para praticar o futebol, parte de sua cultura, e importante vetor de sua experiência (Oliveira, 2020), sem que acatassem todas as tentativas de imposição sobre as práticas esportivas defendidas pela associação. E isso era possível porque, a partir da experiência, novas problemáticas se impõem (Thompson, 1981).

Mesmo não parecendo surtir efeito, a propaganda do ideal higienista de esporte continua forte nas páginas do periódico bancário. Tem início uma campanha para expansão dos horizontes esportivos. Era preciso não parar no futebol. Olhando-se para outras nações era possível ver o atraso do esporte bancário brasileiro. Nos EUA e na Inglaterra eram praticadas todas as modalidades esportivas, “encaixadas regularmente nas diversas estações do ano. Lá, raro é o estabelecimento bancário que não tenha a sua praça de esportes confortavelmente montada”. Até mesmo na Argentina era possível encontrar estabelecimentos de crédito “com seus estádios magnificamente montados, com campos de futebol, de atletismo, quadras de tênis, piscinas etc.” (Não paremos..., abr. 1931, p.6). A LBEA,

com dois anos apenas de vida, arregimenta ela, sob sua bandeira, nada menos que 13 agremiações, representando 15 bancos. Se no futebol é ela forte, por que não poderá ser no cestobol, no atletismo, na natação, no remo etc.? ... Uma vez que temos muitos elementos para tal, atrás dos quais naturalmente acorrerão muitos outros, não se justifica o fato de permanecermos unicamente nos campeonatos de futebol (Não paremos..., abr. 1931, p.6).

O atletismo já fazia sucesso entre os bancários em 1929. Apesar de ainda não ser parte dos esportes patrocinados pela liga, alguns bancários o praticavam no C.A. Paulistano, clube de elite de São Paulo. Esse esporte era assim propagandeado no *Vida Bancária*:

O atletismo praticado racionalmente, isto é, sem excessos prejudiciais, de acordo com o físico das pessoas e acompanhado pela ginástica e exercícios respiratórios, é indubitavelmente um esplêndido esporte para a melhoria do físico e do moral de uma raça. Nele, a lealdade e a polidez nunca cedem os primeiros lugares a outras qualidades, quaisquer que elas sejam (Atletismo, dez. 1929, p.2).

Em 1931 o projeto do campeonato de atletismo finalmente se concretizou. Muitos eram os benefícios que o “esporte base” poderia trazer. De maneira geral,

a corrida a pé, que realiza uma ginástica pulmonar, atraente e eficaz, é um dos exercícios melhores e mais completos, porque é essencialmente estênico, isto é, um exercício que provoca aumento de vigor e atividade e deixa depois do repouso uma respiração fácil, profunda e mais lenta, além de produzir uma sensação sedativa do sistema nervoso e determinar o desenvolvimento torácico, muscular e ósseo (A corrida, dez. 1931, p.4).

Para os bancários, no entanto, os benefícios do atletismo não paravam por aí. O aprimoramento físico aparecia como tão importante quanto o da raça. Yapo (out. 1931, p.5) dizia que “à classe de funcionários de bancos, composta na sua quase totalidade de rapazes de bom preparo e instrução, não escapava o valor do esporte básico, quer na formação das qualidades físicas, quer no enrijecimento das qualidades morais do indivíduo”. Por meio desses excertos é possível, novamente, perceber como os discursos sobre a prática esportiva, nesse caso, sobre o atletismo, estavam em consonância com as ideias de uma perspectiva higienista em um sentido amplo, a despeito da heterogeneidade do higienismo, pois por parte dos organizadores das competições entre os bancários seria uma forma de fomentar hábitos e comportamentos que promoveriam uma melhoria das condições de vida daqueles trabalhadores e, por conseguinte, de sua imagem na sociedade.

Se os benefícios físicos do esporte eram importantes para os bancários que passavam horas do dia fechados nas agências, com má iluminação e pouca ventilação, sem conforto, com pouco espaço e suscetíveis à tuberculose, os benefícios morais não poderiam ser considerados menos importantes do ponto de vista dos organizadores das competições. Era preciso ter moral elevada para ser bancário. Afinal, a posição social ocupada por esses sujeitos exigia deles uma identidade profissional bastante diferente da exigida de outras categorias de trabalhadores. Retidão de caráter, não envolvimento com ilicitudes ou agiotagem e ter sempre boa aparência eram apenas algumas dessas exigências. Desse modo, o discurso de que o esporte ajudava na consagração de um indivíduo física e moralmente melhor cabia muito bem à identidade do bancário. Isso porque a prática esportiva ainda ajudava na interação entre os funcionários – porém, apenas se praticado com cautela; do contrário, poderia gerar conflitos nos locais de trabalho, como demonstrou Fatima Antunes (1992) ao tratar dos operários-jogadores.

## Considerações finais

Até aqui foi possível analisar como a LBEA enxergava a prática esportiva nos meios bancários. Para ela, como explicitado, os esportes eram uma forma de melhorar física e moralmente os trabalhadores bancários, que frequentavam locais de trabalho pouco higiênicos, com pouca iluminação e ventilação, e que tinham grande quantidade de trabalho.

Se não é possível afirmar que essa visão higienista de esporte era consensual entre os esportistas da LBEA, podemos dizer que ela recebia respaldo, pelo menos em parte, dos seus dirigentes, uma vez que eles eram os responsáveis pela seção esportiva do jornal *Vida Bancária*. No entanto, mesmo que esse respaldo existisse no discurso, a prática esportiva fugia do modelo esperado pela diretoria da liga, como foi possível perceber com os casos de consumo de bebida alcoólica pelos atletas. Formava-se, assim, uma cultura esportiva que ia além da prática esportiva pura e simples. Ela se ligava, também, aos momentos de sociabilidade dos bancários, promovendo congraçamento e diversão, contribuindo para a formação de uma cultura de classe.

Esses momentos de sociabilidade eram bastante valorizados entre eles, o que demonstra uma diferenciação entre essa e outras categorias de trabalhadores. Fazendo parte do que se entendia como classe média, seu emprego, assim como suas roupas, seus locais de moradia, sua alimentação e sua ocupação do tempo livre eram diferentes daqueles dos operários, forjando uma identidade profissional.

Há dificuldade em situar esses trabalhadores na esfera social, uma vez que são entendidos pela sociedade na qual se inserem de uma maneira, e se entendem de outra. No entanto, levando em consideração sua identidade profissional, sua faixa salarial e sua vida cultural, com a busca por divertimentos e instrução, pode-se dizer que eles faziam parte da classe média urbana que ganhava força no país no início do século XX. E, em sua busca por divertimentos, elegeram como um dos seus preferidos o futebol, praticado no interior da sua associação de classe. Além disso, apesar do possível interesse dos empregadores para que seus funcionários praticassem esportes – numa tentativa de controle do corpo e do tempo de não trabalho –, o que se percebe na experiência desses sujeitos foi uma ressignificação das práticas, subvertendo a imposição, seja de patrões, seja dos organizadores do esporte bancário, transformando as competições esportivas em momentos de divertimento e congraçamento.

## NOTAS

1 As tabelas foram feitas de maneira manuscrita. Canedo (1977, p.27) as apresenta como se fossem de 1935. Girardi Junior (1995, p.83) as apresenta como produzidas em 1934. As tabelas encontradas no sindicato tinham apenas uma anotação de que foram produzidas na década de 1930, sem mais especificações.

2 Essa é uma média que leva em consideração o salário dos chefes e gerentes, e, por isso, segundo o jornal, é mais alta do que a realidade da maioria dos funcionários de bancos.
